# Neurologists’ perspectives on management challenges and mitigation strategies for Parkinson’s disease patients: A qualitative study in Iraq

**DOI:** 10.1371/journal.pone.0326851

**Published:** 2025-06-26

**Authors:** Mena Khalid Ibrahim, Samer Imad Mohammed, Gheyath Abd Ali Shallal Al-Gawwam

**Affiliations:** 1 Ministry of Health, Baghdad Al-karkh Health Directorate, Baghdad, Iraq; 2 Department of Clinical Pharmacy, College of Pharmacy, University of Baghdad, Baghdad, Iraq; 3 Department of Medical, College of Medicine, University of Baghdad, Baghdad, Iraq; University of Thessaly Faculty of Medicine: Panepistemio Thessalias Tmema Iatrikes, GREECE

## Abstract

**Background:**

Parkinson’s disease (PD) is currently the fastest-growing neurological disorder in the world. Patients with PD face numerous challenges in managing their chronic condition, particularly in countries with scarce healthcare infrastructure.

**Objective:**

This qualitative study aimed to delve into neurologists’ perspectives on challenges and gaps in the Iraqi healthcare system that influence the management of PD, as well as strategies to mitigate these obstacles.

**Method:**

Semi-structured interviews were conducted with neurologists from five different Iraqi provinces, working in both hospitals and private neurology clinics, between November 2024 and January 2025. A thematic analysis approach was employed to identify the main challenges and gaps in the healthcare system, along with potential mitigation strategies for improving PD management in Iraq.

**Results:**

Fourteen neurologists participated in this study. Among them, 71.4% identified medication adherence as a major challenge, followed by motor symptoms (64.3%). Physical dependency due to motor impairment was the most reported limitation affecting daily life and disease management. Neurologists cited several factors influencing patients’ knowledge, including educational background, depression or family support, were mentioned by neurologists. Significant gaps in the Iraqi healthcare system were noted, such as the lack of specialized abnormal movement disorders clinics, multidisciplinary collaboration, a universal healthcare system, insufficient public PD awareness, and the presence of unregulated pharmaceuticals in the market.

**Conclusion:**

Addressing these challenges requires policy-driven reforms, enhanced regulatory oversight and the integration of multidisciplinary care frameworks to optimize the management of individuals with PD. Strengthening patient education and professional training programs could further raise public awareness and improve care quality. Future research should focus on evaluating the effects of these proposed interventions on clinical outcomes for PD patients and promoting more patient-centered care for individuals with PD in Iraq.

## Introduction

Parkinson’s disease, once considered a rare condition, has become highly prevalent in under two centuries, currently recognized as the most rapidly expanding neurological disorder concerning age-standardized prevalence, disability, and mortality rates [1,2]. The clinical presentation of Parkinson’s disease extends beyond the conventional definitions of stiffness, tremors, and bradykinesia, incorporating a variety of non-motor symptoms such as sleep difficulties, gastrointestinal dysfunction, olfactory abnormalities, anxiety, and depressive episodes, which are frequent and contribute to the burden of disease on the patient’s quality of life [3,4]. These symptoms have a significant impact on PD patients’ daily performance and overall well-being [5].

Recent research has highlighted numerous challenges encountered by individuals with PD, such as diagnosis delay [6], complex medical needs, substantial caregiver burden, and suboptimal response to drugs [7], Limited health literacy, unproductive communication, incohesive care coordination, and scarce mental health and rehabilitation services further hinder healthcare accessibility [8]. In the Middle East, individuals with PD experience additional obstacles in their attempt to access adequate care, including limited access to specialized rehabilitation centers and cultural determinants affecting healthcare participation. Additionally, individual characteristics such as inadequate education and poor motivation further impede the successful management of the disease [9].

In Iraq, the management of PD is hindered by insufficient epidemiological data, a shortage of specialized healthcare practitioners, and limited diagnostic capabilities. Community-based research in Baghdad’s Al-Kadhimiya district indicated a crude prevalence of PD at 108.75 per 100,000, with elevated rates in urban regions. In contrast, national estimates from the Global Burden of Disease Study indicated a prevalence of 74.2 per 100,000 in 2019, with an increasing trend over time [10,11]. The healthcare system is profoundly influenced by persistent political instability, declining infrastructure, and socioeconomic difficulties, including poverty and restricted access to potable water and sanitary facilities. The inconsistent availability and costly pricing of medications, along with the absence of a reimbursement framework, impose financial hardship on patients. Diagnostic services and neurologist accessibility are predominantly centralized in urban areas, contributing to disparities in access and intensifying healthcare inequities among regions [12–14].

To successfully address the various challenges encountered by PD patients, collaborative efforts from a multidisciplinary team are essential [15]. Neurologists, who are at the forefront of managing Parkinson’s disease, provide specialized care that is related to enhanced clinical outcomes and survival rates [16].

Their involvement extends beyond treatment to encompass diagnostic accuracy and healthcare accessibility, which are essential for enhanced patient management. Thus, understanding neurologists’ viewpoints is crucial to addressing gaps in diagnosis, treatment, and healthcare approachability. This study explores neurologists’ firsthand experiences and opinions on the primary challenges in managing PD in Iraq and examines potential strategies to mitigate these challenges, offering an in-depth understanding of the barriers that impede optimal patient outcomes.

## Method

### Study design

In this study, we used a qualitative research design to explore neurologists’ perspectives on the barriers and challenges faced by individuals with Parkinson’s disease (PD) in Iraq, as well as mitigation strategies to help overcome these challenges. Semi-structured, open-ended interview questions guided the discussion. Probes were used to elucidate ambiguous responses and to generate more comments when required. The interview guide was generated using artificial intelligence, this was accomplished by inputting data aligned with the qualitative objectives of the study. The guide was reviewed by a panel of experts (five academic clinical pharmacists with experience in qualitative research and one board-certified neurologist). The validated interview guide consisted of seven questions. The first six questions focused on key areas, including daily barriers faced by PD patients, the limitation that prevents them from actively managing their health, the neurologist’s opinion on patients’ knowledge and skill regarding their condition, whether there are any gaps in health care system affecting patients care, technology utilization for PD patients and the effect of socioeconomic factors in disease management. The seventh and final question invited neurologists to share any additional challenges, limitations, and insights they believed were important and that were not covered in the interviews.

### Setting and participant recruitment method

To acquire a comprehensive understanding of neurologists’ insights regarding the barriers and challenges faced by PD patients from multiple clinical and geographical perspectives, interviews were conducted with neurologists from five provinces in Iraq: Baghdad, Najaf, Babel, Fallujah, and Nasiriyah. Neurologists were selected based on a combination of convenience and purposive sampling. Eligible participants were neurologists with at least two years of clinical expertise in managing Parkinson’s disease and a willingness to participate. Neurologists were excluded if they declined to participate, lacked sufficient clinical exposure to patients with PD, or had no practical expertise in disease management.

In Baghdad, nine Interviews were conducted face-to-face in two settings: outpatients and private clinics. To expand the scope of our research and include insights from other regions, five interviews were conducted remotely with neurologists in Najaf, Babel, and Nasiriyah via phone calls. The time and date for each interview were scheduled beforehand with the participants. The current study was ethically approved by the ethical committee at the College of Pharmacy/University of Baghdad (approval number RECO624275). (which functions as the Institutional Review Board [IRB] for research involving human subjects). All participants were explicitly informed about the audio recording and provided verbal consent prior to the commencement of the interviews. To ensure that this procedure is sufficiently documented, the author obtained additional audio-recorded consent. The initial author (Mena Khalid Ibrahim) obtained verbal consent from participants at the beginning of each recording. Participants verbally affirmed their willingness to participate and acknowledged their awareness of being recorded. This method preserves direct evidence of assent.

Subsequently, the second researcher (Samer Imad Mohammed) reviewed the recordings subsequent to the interview to verify that unambiguous consent was obtained and documented. This was done to ensure accountability. This dual-step procedure is in accordance with the ethical standards for minimal-risk studies in which written consent is not practicable.

This approach, which combines real-time aural documentation with independent witness validation, was implemented to preserve transparency while also respecting the autonomy of participants and the pragmatics of the study. We are delighted to provide supplementary information if required.

Verbal consent was chosen instead of written consent due to cultural considerations, as participants from the Iraqi population may feel apprehensive about signing formal documents. The verbal process was more reassuring and acceptable to participants, thus promoting voluntary participation while maintaining ethical rigor.

### Data collection and analysis

Face-to-face interviews were recorded using an iPhone 13 Pro Max, and the interviews conducted remotely via phone calls were recorded using an iPad. Each interview lasted about 12–30 minutes. To achieve the study aims, interviews were conducted until the point of saturation was reached, at which point no new comments were generated. The interviews were continued from November 3^rd^, 2024, to January 30^th^, 2025. Qualitative data categorization was implemented following the manual coding of all interviews. The coding procedure was established by the first study author (MSc candidate in clinical pharmacy) and reviewed by the second study author (PhD in clinical pharmacy). Thematic analysis was conducted using Braun and Clarke’s six steps:“ data familiarization, code generation, integration of codes into themes, revising themes, determining the importance of the themes, and lastly reporting the results.” [17]. QDA Miner Lite software was used for code retrieval and code frequency calculation. To ensure reflexivity and rigor, a reflective journal with all the registered details from interviews was kept throughout the research process to ensure transparency in thematic analysis and alleviate potential researcher bias that may arise during qualitative research.

## Results

**Fig 1:**
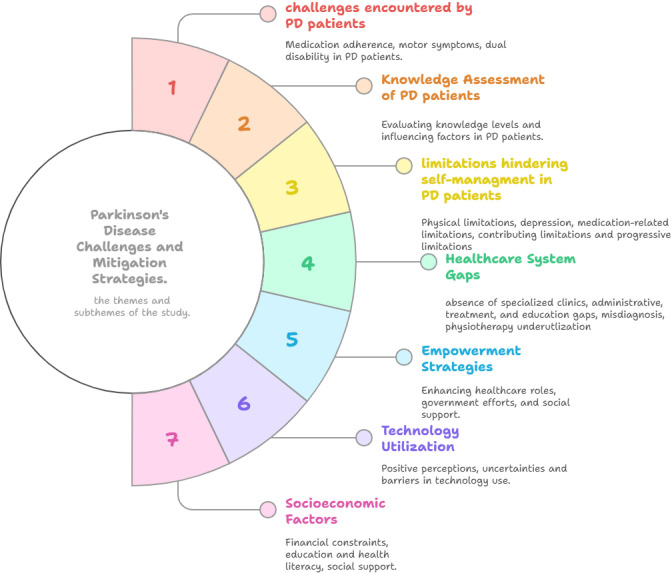
A thematic map illustrating neurologists’ viewpoints on obstacles to patient participation in the management of Parkinson’s disease. The map outlines seven principal themes extracted from qualitative interviews. Each theme is associated with subthemes that arose during thematic analysis.

In this qualitative study, fourteen participants were interviewed. Twelve were board-certified specialists in neurology, while the remaining two were in their final year of the neurology board training program. Most participants (N = 10) were male, and the majority (N = 12) had an Iraqi board in clinical neurology. Nearly half of the participants (N = 6) worked in Baghdad Teaching Hospital. Additional information on research participants is presented in Tables 1 and 2. The resultant study themes and subthemes are outlined in Fig 1.

**Table 1:** The demographics of study participants.

Parameter		Value
**Age (in years) Mean ± SD**		39.14 ± 4.69
**Gender**	Male n (%)	10 (71.4%)
	Female n (%)	4 (28.6%)
**Experience**	Less than 5 years n (%)	2 (14.3%)
	5-10 years n (%)	7 (50%)
	More than 10 years n (%)	5 (35.7%)
**Academic degree**	Board Certified Neurologist n (%)	12 (85.7%)
	Final-year neurology resident n (%)	2 (14.3%)
**Working place**	Baghdad Teaching Hospital n (%)	6 (42.8%)
	Nasiriyah Teaching Hospital n (%)	3 (21.4%)
	Al-Yarmouk Teaching Hospital n (%)	1 (7.14%)
	Al-Numan Teaching Hospital n (%)	1 (7.14%)
	Al-Najaf Al-Ashraf Teaching Hospital n (%)	1 (7.14%)
	Al-Hilla General Teaching Hospital n (%)	1 (7.14%)
	Fallujah Teaching Hospital n (%)	1 (7.14%)

**Table 2:** The characteristics of study participants.

Neurologists	Age	Gender	Working place	Years of Experience	Academic degree
N 1	37	Male	Baghdad Teaching Hospital	5-10 years	Board Certified Neurologist
N 2	48	Male	Baghdad Teaching Hospital	More than 10 years	Board Certified Neurologist
N 3	35	Female	Baghdad Teaching Hospital	Less than 5 years	Final-year neurology resident
N 4	45	Male	Baghdad Teaching Hospital	More than 10 years	Board Certified Neurologist
N 5	37	Female	Baghdad Teaching Hospital	5-10 years	Board Certified Neurologist
N 6	35	Male	Baghdad Teaching Hospital	Less than 5 years	Final-year neurology resident
N 7	38	Male	Al-Hilla General Teaching Hospital	5-10 years	Board Certified Neurologist
N 8	36	Male	Fallujah Teaching Hospital	5-10 years	Board Certified Neurologist
N 9	41	Male	Nasiriyah Teaching Hospital	More than 10 years	Board Certified Neurologist
N 10	46	Male	Al-Yarmouk Teaching Hospital	More than 10 years	Board Certified Neurologist
N 11	48	Male	Al-Numan Teaching Hospital	More than 10 years	Board Certified Neurologist
N 12	36	Female	Nasiriyah Teaching Hospital	5-10 years	Board Certified Neurologist
N 13	36	Female	Nasiriyah Teaching Hospital	5-10 years	Board Certified Neurologist
N 14	38	Male	Al-Najaf Al-Ashraf Teaching Hospital	5-10 years	Board Certified Neurologist

### The challenges encountered by PD patients

The most frequently reported challenge by individuals with Parkinson’s disease (PD) was medication adherence, with neurologists (N = 10) emphasizing that patients struggle with maintaining consistent treatment with anti-parkinsonian drugs due to the expensive nature of these medications (N = 5), their unavailability (N = 3), side effects related to the medications like nausea and dyskinesia due to levodopa (N = 4), drug-drug interactions or drug-food interaction (N = 1), the decreased efficacy of drugs overtime (N = 1).

Additionally, most neurologists (N = 9) highlighted motor symptoms such as (tremors, rigidity, bradykinesia, and shuffling gait) as primary difficulties for PD patients’ daily functioning and quality of life. Few neurologists stated that motor symptoms are the main reason patients with PD first seek a neurologist’s counsel(N = 4) and that they cause social embarrassment for patients when in public (N = 2).

Moreover, few neurologists (N = 5) described a dual burden of disability, where patients experience daily challenges related to both motor and non-motor symptoms such as depression, constipation, REM sleep disorders, and imbalance, further affecting their disease management.

*“Motor symptoms will hinder the patient in his daily activities and job. Most patients with PD are highly educated people like college professors or influential figures. Their voice tone, hand movements, and signature are affected by motor symptoms*” -N7

*“The challenges are multifactorial; the motor symptoms (tremors, bradykinesia, and postural instability) are the biggest challenge for PD patients, but depression is also debilitating for most patients” -N8*

*“Medication adherence can be affected because most drugs are costly, and Patients may be unable to afford drugs, so they might stop using them.”* -N9

### Knowledge assessment of PD patients

The majority of neurologists (N = 12) provided insights regarding the level of knowledge among PD patients. A few neurologists (N = 5) described patients as “ well-informed, highlighting that these patients, compared with other patients, have better compliance with treatment and are more oriented about their disease and drugs. A few neurologists (N=3) declared that most patients have “ poor knowledge” and struggle to comprehend the symptoms and the effects of medications despite receiving explanations of their functions. Other neurologists (N = 3) stated that the information improves with time due to the chronic nature of the disease. A neurologist (N = 1) thought that patients have moderate knowledge (50/50) and possess some knowledge but have gaps in their understanding of the disease.

Several neurologists explained that a patient’s knowledge is influenced by multiple factors, such as the educational background of patients (N = 3), the presence of a support system(N = 3), suffering from depression (N = 2), the stage of the disease (N = 2), unreliable sources of information (N = 1), and having a low socioeconomic background (N = 1).

*“ Patients might be unoriented at first in the early stages when first diagnosed; due to the chronic nature of PD, they become more oriented about their condition later. This is also affected by the education level of patients, the support system they have (because most of them are elderly and depend on the caretaker’s help)” -N5*

*“* *They are somehow informed about their condition, but you can’t say they are well-informed, it’s (50/50). The main issue here is that they sometimes get their information from unreliable sources, like social media” -N10*

### Limitations hindering self-management in PD patients

The majority of neurologists (N = 10) emphasized that physical limitations due to motor symptoms are the main limitations faced by PD patients in their daily lives. Nearly half of the participants stated other contributing limitations hindering self-management in patients, such as lack of support system (N = 2), cognitive impairment (N = 3), old age (N = 1), and insufficient education about PD (N = 1).

A few neurologists (N = 4) declared depression “a non-motor symptom” as one of the limitations faced by patients. Additionally, neurologists highlighted medication-related limitations such as the high cost (N = 3) and unavailability of drugs (N = 1). Two neurologists detailed that limitations progress due to the chronic, progressive nature of PD, stating that initially patients find motor symptoms as the main limitation, later on cognitive impairment becomes challenging for them.

“*Physical dependency caused by physical limitation due to motor symptoms is what hinders most patients because they want to depend on themselves in their daily activities like bathing, for example, but they can’t” -N9*

*“The main limitations are the high cost of drugs, which may cause them to discontinue treatment” -N6*

*“The main limitation for patients is psychological, due to depression, it can cause patients to be less responsive to treatment” -N8*

### Gaps in the healthcare system

Neurologists identified several critical gaps in the Healthcare system that impact the management of PD patients. A majority of neurologists (N = 10) highlighted treatment-related gaps, such as the unavailability of PD medications in outpatient neurology clinics, the high cost of medications (N = 6), the availability of limited pharmaceutical routes of medications (N = 3), providing questionable quality medication (N = 1). More than half of the participating neurologists (N = 8) mentioned administrative obstacles such as the lack of a healthcare insurance system (N = 1), outdated lists of medications in public clinics (N = 1), lack of multidisciplinary collaboration between physicians (N = 2), discrepancy in neurologists’ distribution in outpatient clinics (N = 1), and lack of attention from the government regarding PD patients (N = 2).

Nine neurologists specified insufficient education about PD as a gap in the healthcare system. Half of the neurologists (N = 7) stated that the absence of a specialized abnormal movement disorders clinic in hospitals is another important gap. A few neurologists (N = 5) mentioned misdiagnosis as a result of the absence of diagnostic facilities (N = 2), delay in diagnosis and multiple consultations (N = 2), and PD coexistence with other comorbidities (N = 1). Three neurologists stressed the underutilization of physiotherapy in PD management.

*“There are many gaps in the healthcare system, for instance, the absence of a health insurance system. The lack of adequate health insurance for patients, particularly those with Parkinson’s disease, has a significant impact on their access to treatment. Given the high cost and limited availability of Parkinson’s medications. The provision of free medications, while well-intentioned, can lead to a decline in the quality of drugs and limit patients’ options” -N1*

*“The unavailability of drugs in outpatient clinics or having drugs with questionable quality (brand drugs are superior in PD for patients compared to generic drugs). PD is a chronic condition, and patients need to locate a budget for drugs, and most will not afford it; it’s common for patients to ask the neurologist to select only 2 or 1 drug for them to buy despite their need for more options due to low financial state, this presents a challenge even for the neurologist” -N4*

### Patients’ empowerment strategies

Neurologists emphasized the importance of implementing various strategies to empower PD patients and enhance their abilities to manage their condition. The majority of participating neurologists (N = 13) explained various strategies for the enhancement of the healthcare provider’s role, such as the importance of communication skills with patients (N = 5), providing patients with thorough education about PD (N = 8), tailoring treatment according to the patient’s condition (N = 3), conducting thorough clinical examination (N = 2), encouraging physiotherapy utilization by PD patients (N = 2), staying up-to-date (N = 1), conservative prescribing of medication (N = 1), encourage patients to accept their condition (N = 1).

Twelve neurologists discussed the role of government-led efforts in improving patient empowerment. Key suggestions included the establishment of specialized abnormal movement disorders clinics (N = 7), which could provide multidisciplinary care tailored to patients’ needs, along with providing medication for PD patients in these clinics. Additionally, neurologists advocated for government-supported educational programs to enhance public and patient awareness regarding Parkinson’s disease and its management (N = 5). A few neurologists discussed the importance of implementing physiotherapy in the treatment plan for PD patients (N = 2) and the provision of medication in outpatient clinics (N = 2). One neurologist emphasized the need to foster communication between neurologists and the medication selection committee inside the Ministry of Health.

Three neurologists highlighted the significance of family support in empowering patients, stressing the necessity of instructing and informing family members on how to help their relatives successfully. They noted that a robust support system is vital for PD patients.

*“Encourage patients to seek physiotherapy and occupational therapy and explain their importance in managing disease and enhancing the quality of life for PD patients “ -N4*

*“A neurologist needs to be patient with PD individuals. We need to talk slowly and calmly to ensure they understand every word because most are elderly. Perform a thorough, accurate clinical examination to ensure you have the correct diagnosis. Patients and their caretakers should get enough education about the condition, the neurologist; having good communication skills with patients is essential. With elderly patients, focus on the importance of having a support system to help them navigate their condition” -N12*

### Technology utilization for PD patients

The majority of neurologists (11 out of 14) expressed a positive perspective on the potential benefits of using technology, such as “ wearable devices and smartphone applications,” to benefit PD patients. They highlighted various ways in which it can be used to enhance medication adherence (N = 5), obtain more information about their condition (N = 2), and further help in communicating with neurologists (N = 1).

A smaller subset of participants(N = 3) were uncertain about technology’s practical impact on PD management. Concerns raised in this group are associated with barriers related to patients, such as the age of most patients, level of education and digital literacy, or simply the unfamiliarity of technology interventions in PD patients’ benefits.

*“I don’t know much about technology utilization for PD, I’m not sure how it can benefit them in real life” -N13*

*“Technology can be utilized to the patient’s benefit; some applications have pictures with different emotions, and patients can select the picture that represents their condition or feeling. This is helpful with patients who have slurred speech or very low voices” -N12*

### The influence of socioeconomic factors in PD management

All the participating neurologists agreed unanimously that socioeconomic factors play a crucial role in PD. Three key aspects arose from the discussion, financial constraints, social support and caretaker resources, and finally, education and health literacy.

Most of the neurologists (N = 10) focused equally on both financial constraints and social support and caretaker resources. Financial barriers represented a significant concern among PD patients, affecting medication adherence, access to healthcare services and caretaker services, and overall quality of life for PD patients. Neurologists emphasized the importance of having a support system for PD individuals; they stated that patients with family involvement have better medication adherence, access to healthcare services, confidence, and overall disease management.

A couple of neurologists (N = 2) pointed out the impact of patients’ health literacy and educational background on their PD prognosis. They suggested that PD patients’ low educational level could lead to a limited understanding of their condition and how improving education and awareness about PD among patients can have a positive impact on treatment outcomes.

*“Socioeconomic factors are very important. Patients need the support of family and caretakers in PD. People with good financial status can get help by hiring a stay-at-home nurse or caretaker and can buy their medication, which means better adherence compared to poor people who can’t afford the drugs” -N12*

## Discussion

The findings of this study showed that the majority of neurologists (71.4%) stated medication adherence as the biggest challenge faced by PD patients, attributing this to the financial burden of medications, their unavailability in outpatient clinics, and their side effects. This finding is in line with another Iraqi study that described how the complicated nature of PD therapeutic management can affect patient adherence [18] A Chinese study found that PD patients only moderately follow their prescriptions of anti-Parkinson’s drugs due to their costly nature [19]. The commonalities seen in these findings highlight that the affordability of medication continues to be a worldwide issue in Parkinson’s disease management, specifically in countries that lack a universal healthcare system. However, our work provides granularity by demonstrating that Iraq’s unregulated pharmaceuticals intensify these problems [20]. This indicates that issues of safety and quality of medications can remain a challenge despite their accessibility. It emphasizes that government-led initiatives are imperative to help ensure medication accessibility for individuals with PD.

Additionally, more than half of neurologists (64.3%) highlighted motor symptoms such as (tremors, rigidity, bradykinesia, and shuffling gait) as principal challenges for PD patients’ daily functioning. The physical limitations caused by motor symptoms emerged as the most reported limitations hindering self-management among PD patients (71.4%). This finding is parallel to the existing literature about the substantial influence of motor symptoms on various aspects of Quality of life [21] and its significant role in PD clinical burden [22]. However, 35.7% of participating neurologists explained how the dual disability caused by both motor and non-motor symptoms (NMS) poses a challenge for PD patients. This is already established by another study that discussed how Gut dysfunction, depression, and REM sleep disorders, among many NMS, can be profoundly disabling and can deteriorate the quality of life [3], and health-related quality of life (HRQoL) in PD individuals [23]. Other contributing limitations hindering self-management were reported by neurologists (42.9%), including depression, lack of a support system, and cognitive impairment. These findings are aligned with the results from a recent study that explained how resilient family support can substantially mitigate the impact of depression on quality of life [24], another study that discussed how cognitive alterations can exert a profound and detrimental influence on individuals and their families [25]. In Iraq, PD patients suffering from depression are faced with unique challenges that impede their ability to obtain the mental health services they require. This is attributed to the fact that mental health is considered a culturally sensitive topic, and mental health services such as psychotherapy are scarce. [26]. Addressing these challenges by enhancing public awareness is vital to improving the mental health of Iraqi patients, especially those living with chronic conditions like PD.

Furthermore, the participating neurologists reported medication-related limitations due to the high cost of medications (28.6%). This result aligns with earlier research indicating that people with Parkinson’s disease experience a significant economic burden, characterized by escalating yearly care costs as their condition advances [27].

Most of the neurologists (71.4%) discussed how multiple factors like the educational background, disease stage, and symptoms like depression and the support of family members could influence the knowledge of PD individuals. This is in line with results from another study that demonstrated that PD patients showed superior comprehension of their condition with a long-standing diagnosis [28], and a higher education level [29,30]. In contrast, another study stated that misapprehensions among PD patients were, in fact, unrelated to, among other factors, educational level [31].

Moreover, neurologists discussed the presence of healthcare gaps impacting overall disease management, such as the absence of specialized abnormal movement disorders clinics in most Iraqi hospitals, treatment-related gaps, physiotherapy underutilization, and other administrative obstacles, including the lack of a healthcare insurance system, multidisciplinary collaboration and the presence of unregulated pharmaceuticals in the market. Similarly, a study conducted in the Philippines recognized treatment gaps in the care of PD, such as the absence of universal healthcare coverage, a multidisciplinary collaboration among healthcare providers, and the availability of anti-Parkinson’s drugs (APDS) [32]. Another Iraqi study highlighted the existence of challenges in the pharmaceutical regulations in the country despite the efforts of the Ministry of Health [20]. Nine of the participating neurologists stated that education about PD among the public population is insufficient. This is consistent with the findings of a study that explained how the Middle East’s limited awareness about PD hinders the provision of appropriate care for individuals with PD [30]. Another study described how improving the care of PD patients can be refined by optimizing the education of healthcare providers and patients [33].

Most participating neurologists agreed on the significant impact of socioeconomic factors in PD management and quality of life for patients. This aligns with the existing literature, a scoping review explained how Socioeconomic variables adversely affect medication adherence in PD patients [34]. Moreover, social isolation and insufficient community involvement have been recognized as substantial factors contributing to mental health issues in PD patients. A review indicates that persons who experience social isolation or a deficiency in community connection demonstrate elevated levels of depression, prevalent non-motor symptoms in PD [35]. Depression in Parkinson’s disease is not only debilitating but also adversely affects overall disease management by diminishing motivation for self-care, physical activity, and adherence to medication protocols. In contrast, strong social and communal support has demonstrated a protective impact, according to a study [36], indicating that Parkinson’s disease patients with robust social networks are more inclined to sustain physical activity, which is essential for controlling symptoms like rigidity, balance difficulties, and overall mobility.

Most neurologists (78.6%) in this study had positive perceptions regarding technology utilization for PD patients. This aligns with recent studies that discussed how Innovative technology that assists with ambulation is enhancing patients’ rehabilitation in PD [37], Wearable sensors for PD are advancing, focusing on enhanced usability, AI integration, and extensive testing, which might lead to earlier diagnosis, individualized therapy, and better lives for patients [38].

Finally, neurologists emphasized the necessity of several approaches to empowering patients, including augmenting the responsibilities of healthcare practitioners and promoting government-led initiatives. These findings are similar to other results that emphasize the vital role of such approaches. Recent studies explained how PD necessitates thorough management that requires incorporating physical therapy [15] and indicated that enhanced focus should be directed toward strengthening interdisciplinary collaboration. [39]. An Irish study explained that a specialized movement disorders clinic can provide essential diagnostic and therapeutic services to individuals with PD [40].

Two Iraqi studies, disclosed that the establishment of health insurance could enhance the quality of healthcare services, alleviate the financial strain associated with private sector prices [41], and that the Ministry of Health necessitates augmented funding from the federal government to function efficiently and requires expert support from international health organizations for training and resources [20]. Although pharmacist-led educational interventions have not been specifically examined in patients with PD, evidence from two interventional studies conducted in Iraq indicates that empowering pharmacists as educators can lead to significant improvements in patient outcomes, including enhanced quality of life, reduced complications, and fewer adverse drug reactions. These findings suggest that enhancing the pharmacist’s role as an educator in PD care may significantly impact health outcomes [42,43]. Finally, a qualitative study concluded that enhancing pharmacists’ knowledge helps individuals in effectively managing their medications [44].

A potential limitation of this study is the inability to include neurologists from all governorates of Iraq, which may affect the representativeness of the findings. However, the authors incorporated neurologists from several specialized centers across Iraq to provide a general overview. Another limitation is the absence of patients’ perspectives, which restricts the depth of the findings. While this reflects the original scope of the research, future research should address this gap by incorporating patients’ experiences.

## Conclusion

This study explored neurologists’ perspectives on challenges, gaps in the healthcare system, and limitations faced by PD patients in Iraq. Medication adherence and motor symptoms emerged as key barriers for PD patients. The neurologists highlighted factors affecting the knowledge of individuals with PD, such as educational background and social support. Physical limitations due to motor symptoms were the primary limitation hindering self-management for PD patients. The neurologists also reported depression and medication-related limitations. Gaps in the healthcare system, such as the absence of specialized abnormal movement disorders clinics, and administrative obstacles, for example, the lack of multidisciplinary collaboration and the health insurance system and the presence of unregulated pharmaceuticals, exacerbate these challenges. Addressing these challenges requires policy-driven reforms, improved regulatory oversight of pharmaceuticals, and the integration of multidisciplinary care models tailored to PD management. Enhancing patient education initiatives and professional training programs could also contribute to better disease awareness and management. Future studies could build on these findings by exploring patients’ insights and assessing the impact of proposed interventions on clinical outcomes for PD patients.

## Supporting information

S1 Appendix Interview guide. (DOCX)

## References

[1] Dorsey ER, Sherer T, Okun MS, Bloem BR. The emerging evidence of the Parkinson pandemic. J Parkinsons Dis. 2018;8(s1):S3–8. doi: 10.3233/JPD-181474 30584159 PMC6311367

[2] Feigin VL, Krishnamurthi RV, Theadom AM, Abajobir AA, Mishra SR, Ahmed MB, et al. Global, regional, and national burden of neurological disorders during 1990-2015: a systematic analysis for the global burden of disease study 2015. Lancet Neurol. 2017;16(11):877–97. doi: 10.1016/S1474-4422(17)30299-5 28931491 PMC5641502

[3] Kumaresan M, Khan S. Spectrum of non-motor symptoms in Parkinson’s disease. Cureus. 2021;13(2):e13275. doi: 10.7759/cureus.13275 33728210 PMC7949722

[4] Muslim AT. Non motor symptoms in patients with Parkinson’s disease in Baghdad hospitals. Al-Kindy Col Med J. 2017;13(1):122–7. doi: 10.47723/kcmj.v13i1.141

[5] Jankovic J. Parkinson’s disease: clinical features and diagnosis. J Neurol Neurosurg Psychiatry. 2008;79(4):368–76. doi: 10.1136/jnnp.2007.131045 18344392

[6] Tolosa E, Garrido A, Scholz SW, Poewe W. Challenges in the diagnosis of Parkinson’s disease. Lancet Neurol. 2021;20(5):385–97. doi: 10.1016/S1474-4422(21)00030-2 33894193 PMC8185633

[7] Fabbri M, Coelho M, Garon M, Biundo R, Mestre TA, Antonini A, et al. Personalized care in late-stage Parkinson’s disease: challenges and opportunities. J Pers Med. 2022;12(5):813. doi: 10.3390/jpm12050813 35629235 PMC9147917

[8] Zaman MS, Ghahari S, McColl MA. Barriers to accessing healthcare services for people with Parkinson’s disease: a scoping review. J Parkinsons Dis. 2021;11(4):1537–53. doi: 10.3233/JPD-212735 34308913 PMC8609702

[9] Khalil H, Bajwa JA. Barriers and facilitators in physical rehabilitation for Parkinson’s disease in the Arabian world. Mov Disord Clin Pract. 2015;2(3):227–9. doi: 10.1002/mdc3.12200 30363552 PMC6178601

[10] Mutalib Kareem AA, Niazi AD, Abdullah A. Prevalence of Parkinson’s disease in Al-Kadihymia district (Baghdad city): community-based study. Iraqi J Med Sci. 2005;4(2):179–86.

[11] Khalil H, Chahine LM, Siddiqui J, Salari M, El-Jaafary S, Aldaajani Z, et al. Parkinson’s Disease in the Middle East, North Africa, and South Asia: consensus from the international parkinson and movement disorder society task force for the middle east. J Parkinsons Dis. 2020;10(2):729–41. doi: 10.3233/JPD-191751 32176653 PMC8203232

[12] Al Hilfi TKY. Toward a healthier Iraq. Yale J Biol Med. 2014;87(3):289–97. https://pmc.ncbi.nlm.nih.gov/articles/PMC4144283/. 25191144 PMC4144283

[13] Dewachi O, Skelton M, Nguyen V-K, Fouad FM, Sitta GA, Maasri Z, et al. Changing therapeutic geographies of the Iraqi and Syrian wars. Lancet. 2014;383(9915):449–57. doi: 10.1016/S0140-6736(13)62299-0 24452046

[14] Al-Ashbal MA, Lami FH. Performance evaluation of health houses in Iraq 2021-2022: a descriptive study. J Family Community Med. 2023;30(2):116–22. doi: 10.4103/jfcm.jfcm_362_22 37303842 PMC10252635

[15] Alameer AAH, Alhuraysi MAM, Alhazmi MI, Al-Ghamdi WAM, Alshehri SAA, Maashi SM, et al. Parkinson disease: an overview for the management techniques via physical therapy. JoE. 2024;3(8). doi: 10.62754/joe.v3i8.6023

[16] Willis AW, Schootman M, Evanoff BA, Perlmutter JS, Racette BA. Neurologist care in Parkinson disease: a utilization, outcomes, and survival study. Neurology. 2011;77(9):851–7. doi: 10.1212/WNL.0b013e31822c9123 21832214 PMC3162639

[17] Braun V, Clarke V. Using thematic analysis in psychology. Qual Res Psychol. 2006;3(2):77–101. doi: 10.1191/1478088706qp063oa

[18] Oglah MR, Kasim AA, Hassan BA. Serum level of S100B protein as a biomarker of therapeutic adherence in Parkinson’s disease patients. Iraqi J Pharm Sci. 2024;33(3):161–70. doi: 10.31351/vol33iss3pp161-170

[19] Yi Z, Mao Y, He C, Zhang Y, Zhou J, Feng XL. Medication adherence and costs of medical care among patients with Parkinson’s disease: an observational study using electronic medical records. BMC Public Health. 2024;24(1):1202. doi: 10.1186/s12889-024-18431-y 38689223 PMC11061997

[20] Al-Jumaili AA, Younus MM, Kannan YJA, Nooruldeen ZE, Al-Nuseirat A. Pharmaceutical regulations in Iraq: from medicine approval to postmarketing. East Mediterr Health J. 2021;27(10):1007–15. doi: 10.26719/emhj.21.025 34766327

[21] Kilinc B, Cetisli-Korkmaz N, Bir LS, Marangoz AD, Senol H. The quality of life in individuals with Parkinson’s disease: is it related to functionality and tremor severity? A cross-sectional study. Physiother Theory Pract. 2024;40(10):2213–22. doi: 10.1080/09593985.2023.2236691 37515776

[22] Antonini A, Emmi A, Campagnolo M. Beyond the dopaminergic system: lessons learned from levodopa resistant symptoms in Parkinson’s disease. Mov Disord Clin Pract. 2023;10(Suppl 2):S50–5. doi: 10.1002/mdc3.13786 37637981 PMC10448140

[23] Barone P, Erro R, Picillo M. Quality of life and nonmotor symptoms in Parkinson’s disease. Int Rev Neurobiol. 2017;133:499–516. doi: 10.1016/bs.irn.2017.05.023 28802930

[24] Bae ES, Kang HS. The effect of depression on quality of life in patients with Parkinson’s disease: mediating effect of family function. J Korean Acad Community Health Nurs. 2022;33(1):105. doi: 10.12799/jkachn.2022.33.1.105

[25] Weintraub D, Mamikonyan E. The Neuropsychiatry of Parkinson disease: a perfect storm. Am J Geriatr Psychiatry. 2019;27(9):998–1018. doi: 10.1016/j.jagp.2019.03.002 31006550 PMC7015280

[26] Jumaah ZS, Al-Jumaili AA. The perceptions of general population about mental health services in Baghdad, Iraq: a qualitative study. IJPS. 2023;32(Suppl.):283–90. doi: 10.31351/vol32isssuppl.pp283-290

[27] Ola V, Puri I, Goswami D, Vibha D, Shukla G, Goyal V, et al. Annual cost of care of Parkinson’s disease and its determinants in North India - a cost of illness study with patient perspective. Ann Indian Acad Neurol. 2022;25(4):660–3. doi: 10.4103/aian.aian_779_21 36211186 PMC9540931

[28] Jitkritsadakul O, Boonrod N, Bhidayasiri R. Knowledge, attitudes and perceptions of Parkinson’s disease: a cross-sectional survey of Asian patients. J Neurol Sci. 2017;374:69–74. doi: 10.1016/j.jns.2016.12.063 28104234

[29] Viwattanakulvanid P, Somrongthong R, Vankwani M, Kavita FN, Kumar R. Predictors and level of knowledge regarding Parkinson’s disease among patients: a cross-sectional study from Thailand. Int J Prev Med. 2020;11:25. doi: 10.4103/ijpvm.IJPVM_221_19 32175065 PMC7050216

[30] Abramian S, Tawil S, Akel M, Haddad C, Salameh P. Parkinson’s disease in the Lebanese population: knowledge and attitude scales’ validation and correlates. BMC Public Health. 2024;24(1):3227. doi: 10.1186/s12889-024-20620-8 39567959 PMC11580560

[31] Salinas MR, Chambers EJ, Ho T, Khemani P, Olson DM, Stutzman S, et al. Patient perceptions and knowledge of Parkinson’s disease and treatment (KnowPD). Clin Park Relat Disord. 2020;3:100038. doi: 10.1016/j.prdoa.2020.100038 34316624 PMC8298769

[32] Jamora RDG, Miyasaki JM. Treatment gaps in Parkinson’s disease care in the Philippines. Neurodegener Dis Manag. 2017;7(4):245–51. doi: 10.2217/nmt-2017-0014 28853633

[33] Zaboon Al-Majidi ZA, Lami FH, Hakimi S. Proportion and potential risk factors of poor glycemic control among patients with Type 2 Diabetes mellitus: experience of a tertiary center in Baghdad, Iraq, 2020. J Fac Med Baghdad. 2024;66(2):209–15. doi: 10.32007/jfacmedbagdad.6622070

[34] Gil G, Tosin MHS, Ferraz HB. The impact of the socioeconomic factor on Parkinson’s disease medication adherence: a scoping review. Arq Neuropsiquiatr. 2024;82(2):1–8. doi: 10.1055/s-0044-1779608 38395420 PMC10890916

[35] Al‐Samiry AH, Mohammed SI, Hammed AM, Maryoosh TM. Alone in the mind: review of research examining the link between loneliness and dementia. Med Adv. 2025;3(1):20–7. doi: 10.1002/med4.70005

[36] Kim MY, Baum CM, Connor LT, Chang C-H, Foster ER. Social factors related to participation among people with Parkinson’s disease. Disabil Rehabil. 2024:1–6. doi: 10.1080/09638288.2024.2447379 PMC13552485 40819386

[37] Lee D-H, Woo B-S, Park Y-H, Lee J-H. General treatments promoting independent living in Parkinson’s patients and physical therapy approaches for improving Gait-A comprehensive review. Medicina (Kaunas). 2024;60(5):711. doi: 10.3390/medicina60050711 38792894 PMC11123276

[38] Bougea A. Application of wearable sensors in Parkinson’s disease: state of the art. J Sens Actuator Netw. 2025;14(2):23. doi: 10.3390/jsan14020023

[39] Nassrullah Z, Al-Jumaili AA. Professional challenges facing pharmacists working at public hospitals in an iraqi province: a qualitative study. Iraqi J Pharm Sci. 2023;32(Suppl.):204–13. doi: 10.31351/vol32isssuppl.pp204-213

[40] Yssel J, Casey E, O’Rourke K, Magennis B, Lynch T. The role of a movement disorders clinic. Ir Med J. 2012;105(2):57–9. 22455244

[41] Al-Jumaili AA, Sameer HN. The insights of experienced pharmacists regarding the iraqi health insurance program: a qualitative study. Iraqi J Pharm Sci. 2023;31(Suppl.):131–40. doi: 10.31351/vol31isssuppl.pp131-140

[42] Alkashaf KH, Mohammed SI. Impact of clinical pharmacist-led interventions on short term quality of life among breast cancer women taking chemotherapy. Iraqi J Pharm Sci. 2024;33(4):166–73. doi: 10.31351/vol33iss4pp166-173

[43] Abbas Munaf Z, Mohammed SI, Al-Gawwam GAAS. Impact of clinical pharmacist-led interventions on short term quality of life among multiple sclerosis patients taking disease-modifying therapy. Iraqi J Pharm Sci. 2025;34(1):256–65. doi: 10.31351/vol34iss1pp256-265

[44] Mikhael EM, Jebur NJ, Jamal MY, Hameed TA. Perception, experience, and practice of Iraqi community pharmacists towards customers with substance use disorder. SAGE Open Med. 2024;12. doi: 10.1177/20503121241275472 39280723 PMC11402081

