# Supplementary material for: Neurologists’ perspectives on management challenges and mitigation strategies for Parkinson’s disease patients: A qualitative study in Iraq
Source: PLoS One. 2025 Jun 26;20(6):e0326851. doi: 10.1371/journal.pone.0326851 (PMC12200708; doi:10.1371/journal.pone.0326851)
Supplement: S1 Appendix — (DOCX) [file pone.0326851.s001.docx]

**S1 Appendix: Interview guide.**

**Part 1: the characteristics of the participant.**

| **Age** |  |
| --- | --- |
| **Gender** |  |
| **Academic degree** |  |
| **Working place** |  |
| **Years of experience** |  |

**Part 2: the interview questions.**

1. In your experience, what are the biggest challenges PD patients face in managing their condition on a daily basis? (Prompt: Motor symptoms, non-motor symptoms, medication adherence, emotional well-being)
2. What do you think about the knowledge and skills of PD patients to self-manage their disease effectively? (Prompt: Sources of information, understanding of medication, symptom recognition, communication with healthcare providers)
3. From your observations, what are the main limitations that hinder PD patients from actively participating in self-management? (prompt: Cognitive difficulties, physical limitations, access to resources, support systems)
4. In your opinion, are there any significant gaps in current healthcare practices that could be improved to better support patients' self-management of PD? (prompt: Educational materials, communication strategies, access to specialists, support groups) if yes , explain.
5. How could healthcare professionals like yourself be more effective in empowering PD patients to become active participants in managing their condition? (prompt: Sharing treatment goals, tailoring communication, utilizing technology)
6. Do you think that the use of technology (e.g., wearable devices, medication reminders) can play a role in supporting self-management or the impact of socioeconomic factors on a patient's abilities?
7. Is there anything else you would like to share about the limitations and challenges faced by PD patients in self-managing their condition?
